# Paracheck® rapid diagnostic test for detecting malaria infection in under five children: a population-based survey in Burkina Faso

**DOI:** 10.1186/1475-2875-13-101

**Published:** 2014-03-17

**Authors:** Sekou Samadoulougou, Fati Kirakoya-Samadoulougou, Sophie Sarrassat, Halidou Tinto, Fidèle Bakiono, Issa Nebié, Annie Robert

**Affiliations:** 1 Pôle Epidémiologie et Biostatistique, Institut de Recherche Expérimentale et Clinique (IREC), Faculté de Santé Publique (FSP), Université catholique de Louvain (UCL), Clos Chapelle-aux-champs 30, bte B1.30.13, 1200 Bruxelles, Belgium; 2 London School of Hygiene and Tropical Medicine, London, UK; 3 Institut de Recherche en Sciences de la Santé (IRSS)/Centre Muraz, Bobo-Dioulasso, Burkina Faso; 4 Centre National de Recherche et de Formation sur le Paludisme (CNRFP), Ouagadougou, Burkina Faso

**Keywords:** Malaria, Diagnosis, RDT, Microscopy, Paracheck

## Abstract

**Background:**

Over the past ten years, Rapid Diagnostic Tests (RDT) played a major role in improving the use of biological malaria diagnosis, in particular in poor-resources settings. In Burkina Faso, a recent Demography and Health Survey (DHS) gave the opportunity to assess the performance of the Paracheck**®** test in under five children nationwide at community level.

**Methods:**

A national representative sample of 14,947 households was selected using a stratified two-stage cluster sampling. In one out of two households, all under five children were eligible to be tested for malaria using both RDT and microscopy diagnosis. Paracheck® performance was assessed using miscroscopy as the gold standard. Sensitivity and specificity were calculated as well as the diagnosis accuracy (DA) and the Youden index.

**Results:**

The malaria infection prevalence was estimated at 66% (95% CI: 64.8-67.2) according to microscopy and at 76.2% (95% CI: 75.1-77.3) according to Paracheck®. The sensitivity and specificity were estimated at 89.9% (95% CI: 89.0-90.8) and 50.4% (95% CI: 48.3-52.6) respectively with a Diagnosis Accuracy of 77% and a Youden index of 40%. The positive predictive value for malaria infection was 77.9% (95% CI: 76.7-79.1) and the negative predictive value was 72.1% (95% CI: 69.7-74.3). Variations were found by age group, period of the year and urban and rural areas, as well as across the 13 regions of the country.

**Conclusion:**

While the sensitivity of the Paracheck**®** test was high, its specificity was poor in the general under five population of Burkina Faso. These results suggest that Paracheck**®** is not suitable to assess malaria infection prevalence at community level in areas with high malaria transmission. In such settings, malaria prevalence in the general population could be estimated using microscopy.

## Background

In Burkina Faso, malaria is the main cause of consultations. In 2011, according to statistics from the ministry of health, this disease was responsible for 61.4% of consultations, 77.7% of hospitalizations, and more than three quarters (79.8%) of deaths occurring in health facilities among under five children [1]. Although routine data from health facilities include presumptive diagnosis and may be subject to reporting errors, malaria appears to be a huge burden for individuals, families, and communities in this country.

Early diagnosis and correct treatment are, therefore, a major public health need in this country in order to reduce morbidity and mortality due to malaria. However, the lack of laboratory equipment in primary health care facilities prevents the biological diagnosis of malaria using microscopy. Clinical diagnosis and presumptive treatment of fever are the rule, leading to a large proportion of over diagnosis due to the non-specific symptoms of malaria [2-5]. In rural Mozambique, in 2008, this proportion was estimated to range from 30% to 70% [6]. Improper use of anti-malarials increases the risk of adverse drug reactions and the evolution of drug resistance [7]. Drug resistance is a global issue requiring the development of new effective treatments [8,9]. Scaling up of artemisinin-based combination therapy, a more expensive anti-malarial treatment, also raises concern about the need for biological diagnosis of malaria.

In the last decade, there has been an increase in the number of companies manufacturing malaria Rapid Diagnosic Tests (RDT). A wide range of field and laboratory trials have been conducted to assess the accuracy and effectiveness of RDTs in order to establish their use in endemic areas [10]. Over the past ten years, these tests have played a major role in improving the use of biological malaria diagnosis, in particular in poor-resources settings [11].

In Burkina Faso, the performance of RDT has been largely documented in clinical settings [12-19], but no study has been undertaken nationwide at community level. A recent Demography and Health Survey (DHS) included for the first time the use of RDT to detect malaria infection in children under five years of age. Using these population data, the performance of one of the most widely used RDT has been assessed nationwide in the community.

## Methods

### 2010-2011 Demography and Health Survey

Burkina Faso’s fourth DHS was carried out from May 2010 to January 2011 [20]. A national representative sample of 14,947 households was selected using a stratified two-stage cluster sampling. In one out of two households, all under five children, either residents or visitors (i.e. present in the household the night before the survey), were eligible to be tested for malaria using both RDT and microscopy diagnosis. Absentees were revisited to recruit those missing at the first or second visit. Parents or guardians provided their consent for their child’s participation.

### Malaria diagnosis

At the day of the visit, fieldworkers tested children in their household using a RDT and thick and thin blood films were used by a laboratory technician to assess malaria infection prevalence by microscopy later.

In Burkina Faso, *Plasmodium falciparum* accounts for 95% of malaria infections in children less than five years old [21]. Paracheck® (Orchid Biomedical Systems, Goa, India), which detects a *P. falciparum*-specific HRP-2 protein (PfHRP-2), was used to detect infected children. This test requires approximately 5 μl of blood and is readable after 15 minutes.

Blood slides were labelled and air-dried horizontally in a slide tray. Thin films were fixed with methanol immediately after drying. Slides were stained with 2% Giemsa for 20 minutes in the field. Later, at the reference laboratory (Centre National de Recherche et de Formation sur le Paludisme) in Ouagadougou, two independent technicians read twice each blood slide and classified them as either negative or positive. Another reading was performed by a third microscopist in case of any discrepancy.

### Statistical analysis

Analyses were performed using STATA 12. Prevalence was reported based on RDT results and microscopy. Paracheck® performance was assessed using microscopy as the gold standard. Sensitivity and specificity were calculated as well as the diagnosis accuracy (DA) and the Youden index. DA is the proportion of true results, either positive or negative, among RDT results. It measures the proportion of correct diagnoses and depends on the prevalence of the disease. The Youden index *(J)* is a function of sensitivity *(p)* and specificity *(q)* (J = p + q-1), is not related to prevalence and is commonly used to measure diagnostic performance [22-24]. This index ranges between 0 and 1, with values close to 1 indicating that the performance is relatively large and values close to 0 indicating limited performance.

Results were calculated by age group, urban and rural areas, region, and month of data collection and period of the year. Three periods were defined according to the month of data collection: before the rainy season, in May and June, during the rainy season, from July to October, and after the rainy season, from November to January.

Survey procedures in STATA were used to account for the two-stage cluster sampling as well as the unequal probability of selection between children in rural and urban areas due to weighting. Proportions were compared using a Pearson chi-squared test or a Fischer Exact test. Means were compared using a Student test or Anova and the correlation between prevalence and sensibility or specificity was investigated by a Pearson correlation coefficient.

## Results

6,102 children aged from 6 to 59 months were screened with both Paracheck® test and microscopy during the study period. There were no differences in the mean age (p = 0.93) and the sex distribution (p = 0.98) of recruited children across the 13 regions of the country (Table 1).

**Table 1:** Children mean age and sex distribution across the 13 regions of Burkina Faso

		**Age (months)**		**Boy**		**Girl**	
	**Mean**	**±**	**SD**	**n**	**%**	**n**	**%**
Central	32.5	±	15.3	246	50.0	246	50.0
Boucle du Mouhoun	31.3	±	14.9	384	51.1	367	48.9
Cascades	32.6	±	15.5	113	50.2	112	49.8
Central-East	32.3	±	15.3	252	51.7	235	48.3
Central-North	31.8	±	14.8	260	50.3	257	49.7
Central-West	32.9	±	15.5	252	51.7	237	48.5
Centre-South	32.4	±	15.2	141	53.2	124	46.8
East	32.7	±	15.4	362	52.6	326	47.4
Hauts Bassins	32.8	±	15.3	374	50.3	369	49.7
North	31.8	±	15.9	254	50.9	245	49.1
Central Plateau	31.7	±	15.4	142	49.7	144	50.3
Sahel	32.0	±	15.6	262	47.8	286	52.2
South-West	31.5	±	15.0	139	51.5	131	48.5
**Burkina**	32.3	±	15.3	3181	50.8	3079	49.2

**Figure 1:**
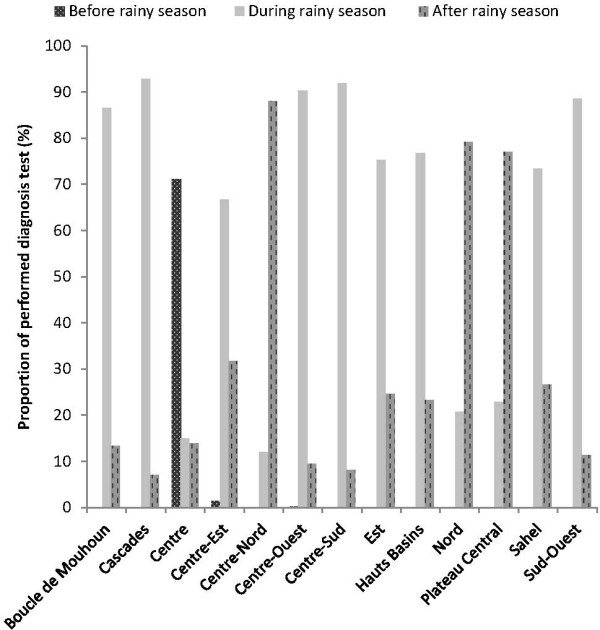
Distribution of diagnostic tests by period of the year in the 13 regions.

Overall, two thirds of the diagnostic tests (62.3%) were performed during the rainy season and slightly less than a third (31.8%) after the rainy season (Figure 1). In three regions – Central-North, Central Plateau and North - tests were mainly performed after the rainy season (87.3%, 76.4% and 82.4% respectively). Before the rainy season, only 5.9% of tests were performed, mainly in the Central region (73.2%).

### Malaria infection prevalence

The overall malaria infection prevalence was estimated at 66% (95% CI: 64.8-67.2) according to microscopy and at 76.2% (95% CI: 75.1-77.3) according to Paracheck®. According to microscopy, the prevalence increased with age group, from 58.6% in 6 to 12 months old children to 69.7% in 48 to 59 months old children (Table 2). By period of the year, the prevalence was only 27.0% (95% CI: 22.0-31.0) before the rainy season while it increased to 69.9% (95% CI: 68.0-71.0) during the rainy season and remained similar after the rainy season, 65.0% (95% CI: 63.0-68.0). In rural areas the prevalence was higher than in urban areas: 73.0% in rural areas versus 18.1% in Ouagadougou.

**Table 2:** Prevalence and performance of Paracheck® by age group, month of data collection and area

		**Microscopy prevalence**	**ST + (n)**	**Sensitivity (%)**	**ST- (n)**	**Specificity (%)**	**DA (%)**	**Youden index (%)**
Area								
	Ouagadougou	18.1	68	80.9	308	73.7	75	55
	Small cities	37.2	242	88.0	408	63.7	73	52
	Rural areas	73.0	3823	90.2	1411	41.5	77	32
	p-value			0.024		<0.001		
Age group (months)								
	6 - 12	58.6	467	89.1	330	54.8	75	44
	13 - 23	61.9	775	89.0	478	52.1	75	41
	24 - 35	66.0	923	91.2	476	54.2	76	45
	36 - 47	70.4	1007	92.3	424	51.7	80	44
	48 - 59	69.7	961	87.4	417	49.2	76	37
	p-value			0.004		0.10		
Month of data collection*								
	May**	20.0	30	93.3	120	73.3	77	67
	June**	31.6	66	83.3	143	69.2	74	53
	July	74.0	376	89.6	132	36.4	76	26
	August	70.9	795	88.6	327	47.1	76	36
	September	73.3	869	88.3	317	44.2	76	32
	October	63.4	688	89.5	398	49.5	75	39
	November	66.3	838	91.8	426	44.6	76	36
	December	64.1	465	93.8	260	59.2	81	53
	p-value***			0.009		<0.001		

*Only seven children were tested in January, **Result for the Central region only, ***Comparison between July and December, ST+: positive blood smear test, ST-: negative blood smear test, DA: Diagnosis accuracy.

The prevalence varied markedly between regions (Table 3). In the Central region, where tests were mainly performed before the rainy season, the prevalence was only 27.6%. Most of regions showed a prevalence ranging between 60 to 70%. The highest prevalence, around 75%, was estimated in five regions – Boucle du Mouhoun, Central-West, Central-South, South-West and Sahel.

**Table 3:** Prevalence, sensitivity and specificity of Paracheck® across the 13 regions

	**Microscopy prevalence**	**Paracheck prevalence**	**ST + (n)**	**Sensitivity (%)**	**ST - (n)**	**Specificity (%)**
Central	27.6	44.1	136	83.8	356	71.1
Boucle du Mouhoun	77.4	90.7	581	95.4	170	25.3
Cascades	60.0	58.2	135	74.1	90	65.6
Central-East	66.9	76.2	326	90.8	161	53.4
Central-North	68.9	84.5	357	95.0	161	39.1
Central-West	76.3	86.1	373	92.5	116	34.5
Centre-South	75.1	92.8	199	97.5	66	21.2
East	69.1	79.3	475	85.9	212	35.4
Hauts Bassins	59.6	67.6	442	90.0	300	65.3
North	64.1	85.6	320	96.6	179	34.1
Central Plateau	61.0	73.8	175	90.3	112	52.7
Sahel	73.7	64.1	404	74.5	144	65.3
South-West	77.8	85.2	210	95.7	60	50.0
**Burkina**	66.0	76.2	4133	89.9	2127	50.4

ST+: positive blood smear test; ST-: negative blood smear test.

### Paracheck® sensitivity and specificity

Tables 2 and 3 summarize Paracheck® performance results. Overall, the sensitivity and specificity were estimated at 89.9% (95% CI: 89.0-90.8) and 50.4% (95% CI: 48.3-52.6), respectively. The positive predictive value for malaria infection was 77.9% (95% CI: 76.7-79.1) and the negative predictive value was 72.1% (95% CI: 69.7-74.3).

By age group, the sensitivity ranged from 89.1% in 6 to 12 months old children to 92.3% in 36 to 47 months old children and slightly decreased to 87.4% in oldest children (Table 2). The specificity ranged from 49.2% to 54.8%.

Sensitivity was high during all of the periods of the study, from 88.3% (95% CI: 81.0-95.6) before the rainy season to 92.8% (95% CI: 91.3-94.3) after the rainy season. Sensitivity was slightly higher in rural areas compared to urban areas. By contrast, specificity did vary markedly by period of the year and area. The highest specificity was estimated to 71.3% (95% CI: 65.6-77.0) before the rainy season and decreased to 44.3% (95% CI: 41.4-47.2) and 51.9% (95% CI: 48.1-55.7) during and after the rainy season respectively. Specificity in urban areas was good but decreased drastically to 41.5% in rural areas.

**Figure 2:**
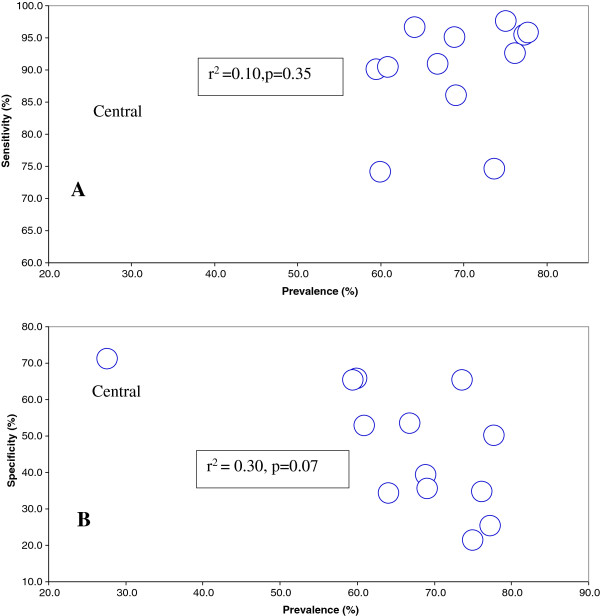
Correlation between sensitivity (A) and specificity (B) of Paracheck® and malaria infection prevalence (microscopy) in the 13 regions.

A large variation across regions was found. The sensitivity ranged from 74.1% in the Cascades region to 97.5% in the Centre-South region (Table 3). While the specificity ranged from 21.2% in Centre-South region to 71.1% in the Central region. Figure 2 shows correlation between the sensitivity and specificity of Paracheck® and malaria infection prevalence in the 13 regions. No significant correlation was found.

### Paracheck® diagnosis accuracy and Youden index

Overall the diagnosis accuracy was estimated at 77% and the Youden index at 40%. The DA did not vary by age group, period of the year or area (Table 2). By contrast, the Youden index was estimated at 71% before the rainy season and dropped to 44% and 52% during and after the rainy season respectively. By rural and urban areas, the Youden index was found higher in urban areas: 55% in Ouagadougou compared to 32% in rural areas.

**Figure 3:**
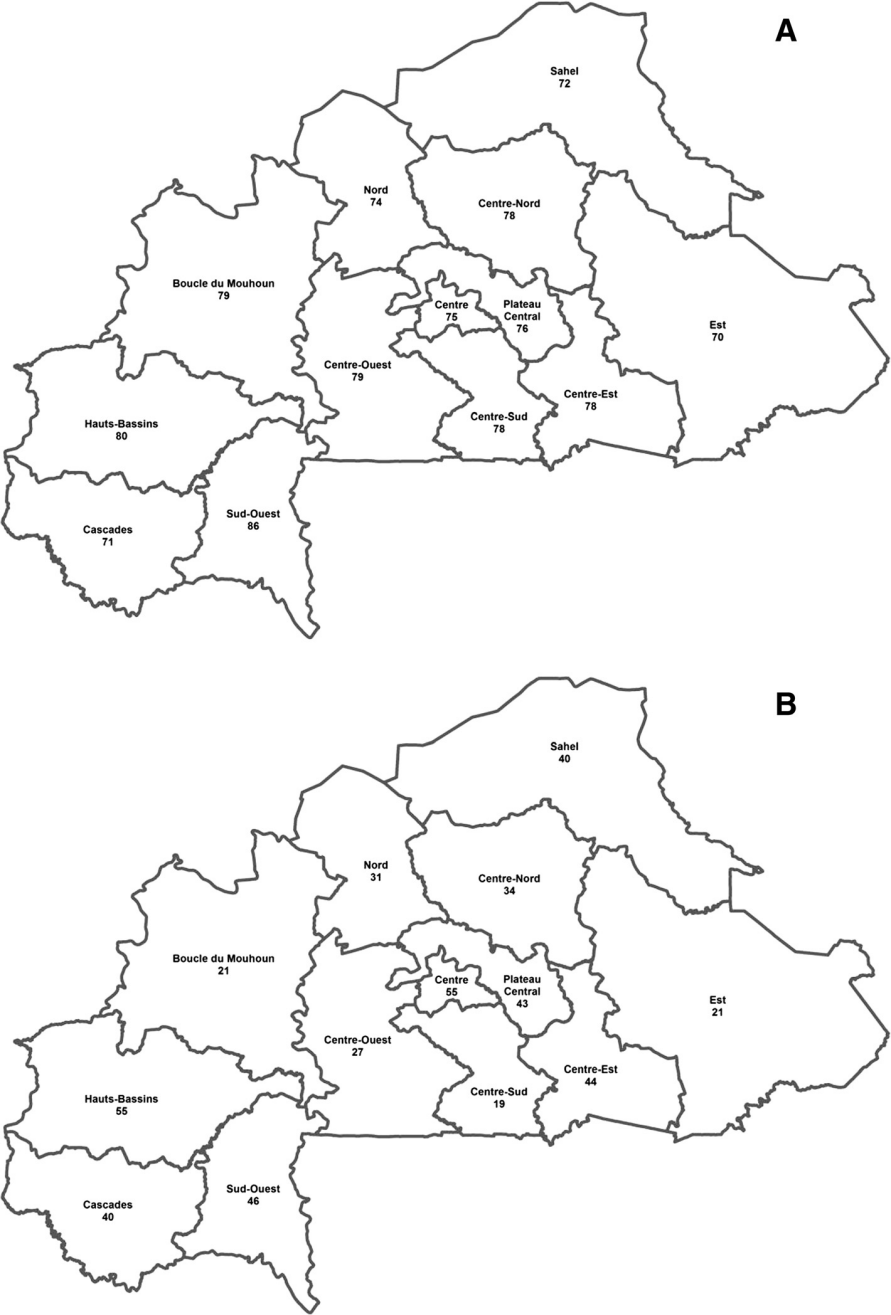
**Diagnosis accuracy (A) and Youden index (B) of Paracheck® in the 13 regions.** The diagnosis accuracy (panel **A**) was quite similar across areas while the Youden index (panel **B**) showed large variations across regions.

A large variation across regions was found, the DA varying from 70% in the East region to 86% in the South-West region and the Youden index from 21% in the East to 55% in both Plateau Central and Hauts-Bassins regions (Figure 3).

## Discussion

The 2010 DHS undertaken in Burkina Faso gave the opportunity to assess the performance of the Paracheck**®** test in under five children nationwide at community level. Overall, the test showed a high sensitivity (89.9%) with a low specificity (50.4%) for the detection of malaria infection. Malaria infection prevalence was high: 66.0% and 76.2% according to microscopy and Paracheck**®** respectively, reflecting an intense transmission of malaria in Burkina Faso, in particular during and after the rainy season.

In other countries, a few studies were undertaken at community level. In Tanzania, in an area of intense malaria transmission, Laurent *et al.* found similar results with a sensitivity and a specificity of the Paracheck**®** test estimated at 96.1% and 63.1% respectively [25]. However, in Ethiopia [26] and Angola [27], while transmission of malaria was low, Pf.HRP-2 RDT showed a low sensitivity, 47.5% and 68.4% respectively, but a high specificity, 98.5% and 92.7% respectively. When the performance is assessed among symptomatic patients, various studies reported a higher specificity of Pf HRP-2 tests. A meta-analysis showed a mean sensitivity of 95% and a mean specificity of 95.2% [28]. In South-West of Burkina Faso, the Paracheck**®** sensitivity and specificity were estimated at 86% and 90% respectively during the dry season, and 94% and 78% respectively during the rainy season [13]. In the Central-West region, its sensitivity and specificity were estimated at 100% and 70.9% respectively [29].

Sensitivity results must be interpreted in relation to parasite density. Indeed, Tiono *et al.* showed that the sensitivity of HRP-2 tests increased with increasing parasite density, from 74.6% when the parasite density was low (0 to 99/μl) to 99.1% when the parasite density was higher (1,000 to 4,999/μl) [30]. This result is supported by other studies using OptiMAL RDT [16] as well as various other tests [10]. Although parasite densities were not available in the 2010 DHS dataset used in this study, these findings could explain why the Paracheck**®** sensitivity was found higher in rural areas, where transmission is higher than in urban areas, and slightly higher in younger children with no or a low level of acquired semi-immunity.

With 50% specificity for the Paracheck**®** test, about half of the children with a positive RDT result had a negative blood slide. This high proportion may be related to the well-known limitation of Pf HRP-2 tests which detect persistent HRP-2 antigenicity in the bloodstream for a few weeks after previous infections [17,25,31]. In addition, RDTs are known to detect low parasite densities (<100/μL) [32] which may not be detected by microscopy [33]. Low parasite densities in asymptomatic carriers are likely to be common at community level, in particular in endemic areas. In Senegal, among 8,636 cases of parasitaemia detected in the general population over a four-month follow-up period during the rainy season, only 4% suffered from fever [34]. In Burkina Faso, during the study period, an episode of fever during the two weeks prior to the interview was reported for 20.6% of children [26]. The malaria infection prevalence being estimated at 66%, this proportion may reflect a relatively large proportion of asymptomatic carriers. Before the rainy season or in Ouagadougou, while the proportion of asymptomatic carriers is likely to be lower, a highest specificity was found, estimated at 71.3% and 73.7% respectively. Laurent *et al.* also showed an increase in specificity with decreasing prevalence [25].

Specificity, as well as positive predictive value, results should however be interpreted bearing in mind one limitation of this study. Indeed, due to the capacity of the test to detect both low parasitaemia and persistent HRP2 antigenicity existing at community level, the discordant cases (positive RDT result/negative microscopy) could not be classified accurately in the absence of a second gold standard such as Polymerase Chain Reaction (PCR).

Variations in the sensitivity, specificity and Youden index were found across the 13 regions of Burkina Faso, whereas diagnosis accuracy was 70% or above. This is certainly due to the difference in the distribution of month of data collection and therefore differences in the transmission level, prevalence and parasite density. While tests were mainly performed before the rainy season in the Centre region, the prevalence was the lowest and the specificity the highest. In addition, under field condition, the performance of RDTs may vary from one user to the other and the quality of the tests may be affected by temperature, humidity, substandard transport or storage condition [35].

## Conclusion

While the sensitivity of the Paracheck**®** test was high, its specificity was poor in the general under five population of Burkina Faso. These results suggest that Paracheck**®** is not suitable to assess malaria infection prevalence at community level in areas with high malaria transmission. In such settings, malaria prevalence in the general population should be estimated using microscopy. However, in other countries where elimination of malaria is targeted, detecting malaria infection at community level, even at very low parasite density levels, is certainly useful.

## Abbreviations

DA: Diagnostic accuracy; DHS: Demography and Health Survey; Pf.HRP-2: *P. falciparum* specific HRP-2 protein; PCR: Polymerase chain reaction; RDT: Rapid diagnostic test; WHO/FIND: World Health Organization/ Foundation for Innovative New Diagnostics.

## Competing interests

The authors declare that they have no competing interests.

## Authors’ contributions

SS (first author) and FKS conceived the study, performed the statistical analysis and drafted the manuscript. SS (third author), HT, and FB contributed to the manuscript by giving substantial intellectual content. IN supervised the fieldwork and carried out microscopy diagnosis. AR contributed to intellectual content, supervised the statistical analysis and the writing up of the manuscript. All authors read and approved the final manuscript.
